# Home-Based Exercise Training in Childhood-Onset Takayasu Arteritis: A Multicenter, Randomized, Controlled Trial

**DOI:** 10.3389/fimmu.2021.705250

**Published:** 2021-07-28

**Authors:** Camilla Astley, Gleice Clemente, Maria Teresa Terreri, Camila G. Carneiro, Marcos S. Lima, Carlos Alberto Buchpiguel, Hilton Leão Filho, Ana Lúcia de Sá Pinto, Clovis Artur Silva, Lucia Maria Arruda Campos, Nadia Emi Aikawa, Saulo Gil, Rosa Maria Rodrigues Pereira, Hamilton Roschel, Bruno Gualano

**Affiliations:** ^1^Applied Physiology & Nutrition Research Group, Laboratory of Assessment and Conditioning in Rheumatology, Faculdade de Medicina da Universidade de São Paulo (FMUSP), Universidade de Sao Paulo, São Paulo, Brazil; ^2^Division of Pediatric Rheumatology, Department of Pediatrics, Federal University of Sao Paulo, São Paulo, Brazil; ^3^Laboratory of Nuclear Medicine (LIM-43), Department of Radiology and Oncology, Hospital das Clínicas da Faculdade de Medicina da Universidade de São Paulo (HCFMUSP), Faculdade de Medicina, Universidade de Sao Paulo, São Paulo, Brazil; ^4^Radiology Institute, Hospital das Clínicas da Faculdade de Medicina da Universidade de São Paulo (HCFMUSP), Faculdade de Medicina, Universidade de Sao Paulo, São Paulo, Brazil; ^5^Rheumatology Division, Hospital das Clínicas da Faculdade de Medicina da Universidade de São Paulo (HCFMUSP), Faculdade de Medicina, Universidade de Sao Paulo, São Paulo, Brazil; ^6^Pediatric Rheumatology Unit, Children’s Institute, Hospital das Clinicas HCFMUSP, Faculdade de Medicina, Universidade de Sao Paulo, São Paulo, Brazil

**Keywords:** vasculitis < rheumatic diseases, arterial inflammation, cardiovascular risk, physical activity, physical exercise

## Abstract

**Introduction:**

Childhood-onset Takayasu Arteritis (c-TA) is a rare, large-vessel vasculitis seen in children that could predisposing patients to a high risk of mortality. Exercise has the potential to improve overall health in several diseases, but evidence remains scant in c-TA. The main objective of this study was to investigate the safety and potential therapeutic effects of exercise in c-TA.

**Methods:**

This was a 12-week, multicenter, randomized, controlled trial, to test the effects of a home-based, exercise intervention *vs*. standard of care in c-TA patients in remission. The primary outcomes were arterial inflammation, assessed by [^18^F] FDG- PET/MRI and systemic inflammatory markers. Secondary outcomes included, physical activity levels, functionality, body composition, disease-related parameters, and quality of life.

**Results:**

Thirty-seven patients were assessed for eligibility, which represents the total number of c-TA patients being followed by the three specialized medical ambulatory services in Sao Paulo. After exclusions, fourteen c-TA patients (71.4% females) aged 12-25 years were randomly allocated into exercised (n=5) and non-exercised groups (n=9). Exercise did not exacerbate arterial inflammation. In fact, exercised patients had a reduction in the frequency of vessel segments with severe inflammation, whereas the non-exercised patients had an opposite response (*P*=0.007). Greater improvements in visceral fat, steps per day, functionality and physical component SF-36 were observed in the exercised patients (*P ≤* 0.05).

**Conclusions:**

Exercise is safe and may improve visceral fat, physical activity levels, functionality, and physical component SF-36 in c-TA patients. Thus, exercise arises as a novel, evidence-based intervention to improve general health in c-TA.

**Clinical Trial Registration:**

https://www.clinicaltrials.gov/ct2/show/NCT03494062?term=NCT03494062&draw=2&rank=1, identifier NCT03494062.

## Highlights

✓ Exercise has the potential to improve inflammation and cardiovascular health in several clinical conditions, but evidence remains scant in c-TA.
✓ Home-based, exercise training program is safe and may improve arterial inflammation and some cardiometabolic risk factors in this population.
✓ These findings demonstrate that exercise may play a relevant therapeutic role in the treatment of c-TA by mitigating symptoms inherent to the disease.

## Introduction

Takayasu Arteritis (TA) is a rare granulomatous, chronic large-vessel vasculitis that involves mostly the aorta and its major branches (1, 2), and it is associated with increased expression of IL-8, IL-12, IL-17, IL-18 or IFN-alfa (3). Vascular inflammation, the main characteristic of disease can lead to stenosis, occlusion, dilatation or aneurysm formation during the course of the disease (4), resulting in a high mortality rate (3 to 15% in 10 years) and impaired quality of life (5).

Cardiovascular disease associated with disease activity has been recognized as the main cause of morbidity and mortality in TA (5), and anti-inflammatory treatment has been suggested as a promising strategy to prevent cardiovascular progression and morbidity in this disease (6). Childhood-onset TA (c-TA) is a still more enigmatic disease, as clinical manifestations in pediatric patients are less specific than in adults, with a mortality rate as high as 35% (7). Current treatment options also aim to attenuate inflammation; however, to the best of our knowledge, there has been no randomized controlled trial (RCT) showing the efficacy of these drugs in pediatric population, with retrospective studies showing patients seem unresponsive to conventional therapies (8).

Exercise has been shown safe and capable of alleviating inflammation in several cardiovascular and autoimmune diseases (9–11) through mechanisms associated with reduction in visceral fat, transient secretion of anti-inflammatory “myokines”, such as IL-6, followed by IL-10 and receptor antagonist of IL-1 (IL-1ra), and/or suppression of toll-like receptors (12). In a quasi-experimental study involving adults with TA, we showed that exercise was able to reduce serum TNF-α and increase soluble TNF receptor 1 (sTNFR1), which was paralleled by increases in angiogenic factors and physical function (10). While exercise can be a treatment in several diseases, its therapeutic role in c-TA remains to be determined.

In this study, we performed a multicenter RCT to investigate the safety and potential therapeutic effects of a home-based, exercise training program on arterial and systemic inflammation, cardiometabolic risk factors, functionality, and quality of life of individuals with c-TA. We hypothesized that exercise would be safe and of potential clinical benefit in c-TA.

## Material and Methods

### Study Design and Patients

This was a 12-week, three-center, RCT conducted between April 2018 and August 2019 in Sao Paulo, Brazil. The study was approved by the ethics committee and registered at ClinicalTrials.gov (NCT03494062). Patients and parents provided written informed consent before entering the study. The manuscript is described according to the CONSORT guidelines.

Patients with c-TA were recruited from the Division of Rheumatology of the School of Medicine and the Pediatric Rheumatology Unit of the Children and Adolescents’ Institute of the University of Sao Paulo, and from the Division of Pediatric Rheumatology of the Federal University of Sao Paulo. Inclusion criteria were patients who fulfilled the current classification for c-TA (13). Of note, these 3 centers represent the total of specialized ambulatory care services for c-TA patients in Sao Paulo, the largest city of Latin America.

Exclusion criteria were pregnancy, heart failure, renal failure, cardiac, pulmonary or musculoskeletal disorders that precluded exercise training, acute infection in the last 30 days and active disease in the last six months based on the Indian Takayasu Arteritis Score 2010 (ITAS2010) (14) and on the Paediatric Vasculitis Activity Score (PVAS) (15). The medication was not modified during the follow-up.

Participants were randomly assigned (2:1) into an exercise (ET) or a control group. After completion of the baseline evaluations, using a computer-generated randomization code for block of three patients. Primary outcomes were arterial inflammation, assessed by [^18^F] fluoro-deoxy-D-glucose positron emission tomography/magnetic resonance imaging [FDG-PET/MRI]) and systemic inflammation markers. Secondary outcomes included physical activity levels, functionality, body composition, disease-related parameters, and quality of life.

### Exercise Training Session

The home-based exercise program consisted of a 12-week aerobic and bodyweight exercise training program following the recommendations by the Canadian Society for Exercise Physiology (16). Training sessions were divided in two parts. The first one was the warm-up, which includes predominantly aerobic exercises such as jumping jack, skipping and mobility and flexibility exercises. The second part included bodyweight exercises for the major muscle groups, such as squats, lunges, push-ups, crunches, and planks. We have selected exercises for upper limbs, lower limbs and core, and the sets were divided into 1-2 exercises for each muscle group. The control group received standard of care. Prior to exercise training, ET received a booklet and video containing instructions on the exercise protocol and participated in a supervised training session led by a fitness instructor, in order to provide them with practical advice on the exercise protocol. Every 4 weeks, patients returned to our laboratory to receive individual information on training progression. Patients and parents also received supportive phone calls and/or text messages every 2 weeks to check the compliance and elucidate any potential questions related to the protocol.

The intervention progressed every 4 weeks, with increases in number of sets, repetitions and/or exercise duration, and less rest between sets. Patients were instructed to exercised three times a week and were advised to report adherence and exercise-related adverse events and symptoms to the protocol on a session basis using a log. The additional video 1 (https://figshare.com/articles/media/VideoS1_Astley_2020_mp4/13341644) displays the details regarding the exercise training program.

### [^18^F] FDG PET/MRI

Images were acquired in a hybrid PET/MRI (GE healthcare Signa PET/MRI 3-Tesla) device, which allows simultaneous evaluation of MRI images co-registered with PET. Patients were instructed to fast for at least 6 hours prior to the injection of [^18^F] FDG. Blood glucose measurement was carried out for all patients before the injection. After the intravenous administration of 185 to 370 MBq of [^18^F] FDG, the patients rested for approximately 60 minutes prior to the imaging acquisition. After planning the number and location of the different bed positions (BP) by the locator images, the combined acquisition of PET/MRI started with an estimated acquisition time of 2 to 4 minutes per BP. In each BP, a volumetric T1 sequence (LAVA FLEX^®^) was obtained (repetition time/echo time [TR/TE] of 4 ms/1.7 ms, slice thickness of 5.2 mm, 256 × 128 matrix), used for attenuation correction purposes.

The following sequences were also performed: T2 with fat suppression (STIR) (TR/TE/inversion time (TI) 1200 ms/42 ms/190 ms, 8 mm slice thickness, 380 × 224 matrix), to evaluate potential edema in the vessel wall; axial diffusion with values ​​of b of 0, 400 and 1000 s/mm^2^ (Matrix of 160 x 160 and 5 mm of thickness); T1 (LAVA^®^) volumetric sequences before and after 15 minutes of intravenous contrast injection, to assess vessel wall thickness and late enhancement.

A dynamic angiography was performed including the proximal third of cervical vessels and extending to the common iliac arteries (longitudinal FOV of 50 cm), using a dedicated time resolved technique (TRICKS^®^), before and after contrast injection in the coronal plane. This technique allows for accurate morphological evaluation of the vessels, with multiple acquisitions at very high temporal resolution, using volumetric acquisitions (TR/TE minimum and 3.9 ms/cut thickness: 3 mm/Matrix: 384 x 192 mm). MRI images were evaluated by an experienced abdominal radiologist (H.L. MD), and PET imaging were assessed by an experienced nuclear medicine physician (M.L. MD), both blinded to the intervention.

The areas of vessel thickening were evaluated morphologically and functionally. The following parameters were evaluated: the measured thickness of the vessel, apparent diffusion coefficients (ADC) values calculated ​​by monoexponential regression, using regions of interest (ROI’s) positioned in the areas abnormal thickness; and the degree of wall’s enhancement using the late contrast series, following normalization with the same region in the pre-contrast phase, using the equation:

(EPos−EPre)/(EPre)

where *EPos* is the vessel signal in the late post-contrast phase, and *EPre* is the signal in the pre-contrast phase. Images were acquired in a hybrid PET/MRI (GE healthcare Signa PET/MRI 3-Tesla) device, which allows simultaneous evaluation of MRI images co-registered with PET. Visual analysis of [^18^F] FDG uptake was performed by the same trained radiologist blinded (M.L., MD) to the intervention in the following segments: right and left carotid artery, right and left vertebral artery, ascending aorta, aortic arch, descending aorta, brachiocephalic artery, right and left subclavian artery, pulmonary trunk, celiac trunk, superior mesenteric trunk, abdominal aorta, right and left renal artery, inferior mesenteric artery and right and left iliac artery. Vascular uptake was graded using the maximum standardized uptake value (SUV_max_), defined as the ratio of [^18^F] FDG activity to injected activity and normalized to the body weight. 3D regions of interest (ROIs) were drawn around the vessel wall in axial, sagittal and coronal slices and the corresponding SUV_max_ was calculated for each aforementioned vessel segments and compared between groups (see **Table 3**).

A visual scoring system was also carried out to quantify the large-vessel [^18^F] FDG uptake as follows: 0, no FDG uptake; I, low-grade uptake (less than liver); II, intermediate-grade uptake (uptake values between those of blood pool and below liver); III, high-grade uptake (greater than liver). The percent distribution of vessel segments with grade III, as a surrogate of arterial inflammation (17), was compared between groups.

### Blood Sampling

After 12-hour overnight fasting, 40 mL of blood was extracted into vacutainer tubes and stored for subsequent analysis of fasting glucose and insulin, blood lipids (low density lipoproteins [LDL], very-low-density lipoproteins [VLDL], high-density lipoprotein [HDL]), total cholesterol, triglycerides, anti- and pro-inflammatory, and angiogenesis markers [i.e.; C-reactive protein (CRP); erythrocyte sedimentation rate (ESR); interferon gamma (IFN-g); interleukin 10 (IL-10); interleukin 12p70 (IL-12p70); interleukin 1 receptor antagonist (IL-1ra); interleukin beta (IL-1b); interleukin 6 (IL-6); tumor necrosis factor alfa (TNF-a); vascular endothelial growth factor (VEGF); platelet-derived grown factor (PDGF)].

Triglycerides, HDL, and CRP were assessed by colorimetric enzymatic methods (CELM, Sao Paulo, Brazil), and LDL and VLDL were calculated following a previous description (18). IFN-γ, IL-10, IL-12p70, IL-1ra, IL-1β, IL-6, TNF-α, VEGF and PDGF were analyzed in a 96-well plate utilizing a kit of 8-cytokine Milliplex MAP Human Cytokine/Chemokine Magnetic Bead Panel (Millipore Corp., Billerica, MA) following the kit-specific protocols.

### Body Composition

Participants underwent a whole-body dual-energy x-ray absorptiometry (DXA) scan (GE Healthcare^®^, WI, USA) to quantify fat mass, percentage fat mass, lean mass, and visceral adipose tissue using CoreScan™ software (enCORE version 17). DXA measurements were carried out by the same trained technician blinded to the intervention.

### Physical Activity Levels and Functionality

Physical activity was objectively measured using Actigraph GT3X accelerometers. All participants were instructed to wear the accelerometer during waking hours, except when bathing or swimming, for 7 consecutive days at the baseline period and after 12-wk intervention. The participants accumulated at least 10 hours of valid activity recordings per day for at least 4 days. The accelerometer was worn on an elastic belt at the waistline on the right side of the hip. Participants were instructed to complete a daily time diary to record when the device was worn and removed to ensure data accuracy (e.g., to distinguish sedentary time from non-wear time). Data were exported from the device every 15-seconds for children and adolescent and, 60-seconds for adults using ActiLife 6 software. Non-wear time was defined as a minimum of 60 minutes of continuous zero counts and days with at least 600 minutes of wear time were considered valid. Freedson cut points were used to define epochs for patients with c-TA aged ≥ 18 years: sedentary time (<100 counts/minute), light-intensity physical activity (≥100 to <1,952 counts/minute), and moderate-to-vigorous physical activity (MVPA) (≥1,952 counts/minute) (19). Evenson cut points were used to define epochs for patients with c-TA aged < 18 years: sedentary time (<100 counts/minute), light-intensity physical activity (≥100 to <2,296 counts/minute), and MVPA (≥2,296 counts/minute) (20). All participants had valid accelerometer data (a minimum of any four valid days). Data are shown as %/day in each domain of intensity.

Muscle function was assessed by Timed-stands (TS) (21) and Timed-up-and-go tests (TUG) (22). One familiarization trial was performed at least 48 hours prior for both tests. The timed-stands test counts the number of stand-ups that a subject is able to perform from a standard height (i.e., 45 cm) armless chair within 30 seconds and the TUG test evaluates the time required to rise from a standard arm chair, walk to a line on the floor 3 meters away, turn, return, and sit down again. Volunteers performed two maximum attempts with 2 minutes interval between them.

### Disease-Related and Health-Related Quality of Life Parameters

Age at disease onset, time since diagnosis, current and cumulative dose of medications were obtained through review of medical records and interviews. Two physicians blinded to the intervention performed the clinical assessments using the following tools: ITAS2010 (14), PVAS (15), and Vasculitis Damage Index (VDI) (23). Health-related quality of life was assessed using the 36-item short-form questionnaire (SF-36), including the domains: functional capacity, physical appearance, pain, general health status, vitality, social aspect, emotional aspect, mental health, and comparative state of health. SF-36 provides a score from 0 to 100, and increasing values indicate better condition (24).

### Sample Size and Statistical Analysis

**Figure 1:**
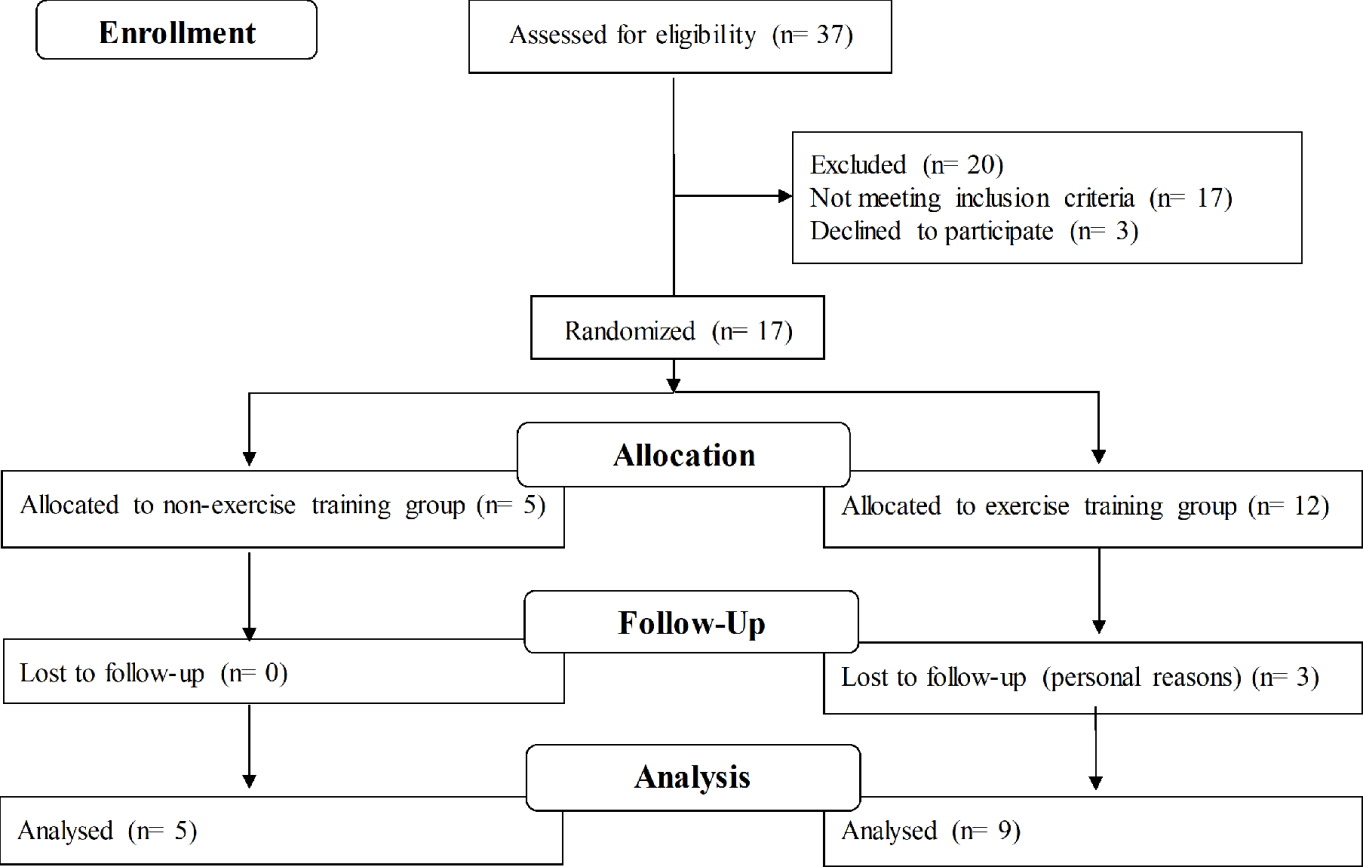
Flow diagram.

As there is a lack of data available for sample size determination, the sample size was chosen based on feasibility, particularly the patients’ availability, in line with current recommendations (25). Due to the rarity of the disease, a sample of 12 c-TA patients (out of 37 existing patients; see *Results* and **Figure 1**) was enrolled.

Data are presented as mean or median, standard deviation (SD) or interquartile range (IQR 25-75), pre-to-post changes (delta), estimated mean difference (EMD), and 95% confidence interval (95%CI), unless otherwise indicated. Comparisons between groups at baseline were performed by Two-tailed unpaired T-test or Fisher exact test. We carried out a complete-case analysis using baseline values as covariate (whenever between-group differences were found at baseline) to test for possible between-group differences in delta values using mixed models using SAS software version 9.1. Possible between-group differences in the frequency of arterial inflammation (grade III) were tested by Fisher exact test. Disease-related parameters and quality of life were analyzed using Friedman’s test for repeated-measures, followed by Wilcoxon test. Significance level was set at *P ≤* 0.05.

## Results

Thirty-seven patients were assessed for eligibility. This represents the total number of patients with c-TA being followed by the three specialized medical ambulatory services in Sao Paulo. After exclusions (17 did not achieve inclusion criteria and 3 declined to participated for personal reasons), fourteen volunteers (71.4% females) aged 12-25 years, with median disease duration of 10 years were recruited and randomly allocated into control group (n=5) or ET (n=9). All patients fulfilled the classification for c-TA (14) and all patients had inactive disease based on disease activity scores previously described (13, 15) Three patients dropped out from ET due to personal reasons (**Figure 1**).

**Table 1**, shows the main patients’ characteristics at baseline. Despite an overall well-controlled disease based on ITAS and PVAS scores, 15 patients (88.2%) showed arterial inflammation (grade III) in at least one of the vessels assessed.

**Table 1:** Baseline, demographic, and disease-related parameters in childhood-onset Takayasu arteritis (c-TA).

	Control(n=5)	Exercise training(n=9)	*P*
*Demographic parameters*			
Age (years)	20.4 (3.21)	17.1 (3.72)	0.822
Height (m)	1.68 (0.16)	1.57 (0.05)	0.003
Body weight (kg)	58.7 (13.5)	55.2 (10.4)	0.486
BMI (kg/m^2^)	20.4 (1.96)	22.2 (3.39)	0.257
Female, n (%)	2 (40)	8 (88.8)	0.094
Disease time (years), n (%)	12.6 (2.97)	8.56 (3.17)	0.963
Age at the disease onset (years)	7.80 (5.07)	8.56 (3.17)	0.420
Arterial hypertension, n (%)	3 (60)	9 (100)	0.109
Infliximab, n (%)	0 (0)	2 (22.2)	0.505
Adalimumab, n (%)	1 (20)	1 (11.1)	1.000
Leflunomide, n (%)	2 (40)	0 (0)	0.505
Methotrexate, n (%)	1 (20)	5 (55.5)	0.307
Prednisone, n (%)	1 (20)	1 (11.1)	1.000
ITAS score (a.u.)	0.00 (0.00-0.50)	0.00 (0.00-0.50)	N/A
PVAS score (a.u.)	0.00 (0.00-1.00)	0.00 (0.00-0.50)	N/A
VDI score (a.u.)	2.00 (2.00-2.50)	1.00 (2.00-2.50)	N/A
SUV grade I	47.3 (21.9)	32.5 (24.9)	0.907
SUV grade II	44.7 (22.1)	56.1 (27.1)	0.798
SUV grade III	7.9 (6.8)	11.2 (9.9)	0.579

Data are expressed as mean (SD), n (%) or median (IQR 25-75). BMI, body mass index; ITAS, indian takayasu's arteritis activity score 2010; PVAS, Pediatric Vasculitis Activity Score; VDI, vascular damage index; SUV, standard uptake value.

The compliance to the exercise training program was 93 ± 0.06%. No adverse events were reported during and after the intervention period. Data on blood inflammatory markers, body composition, physical activity levels, muscle function and cardiometabolic risk factors comparing ET and control groups can be found in **Table 2**. ET showed a small reduction in IL-1β compared with control after the intervention (EMD: -1.76; 95%CI: -3.57, 0.03 pg/mL; *P=*0.053). There were no other changes in blood inflammatory markers. ET had a greater reduction in visceral fat following the intervention (EMD: -0.22; 95%CI: -0.01, -0.44 g; *P=*0.040). The remaining body composition parameters did not differ between groups. Furthermore, ET had a tendency to increase light-intensity physical activity (%/day) and steps count per day, respectively (EMD: 6.82; 95%CI: 13.8, -0.22%/day; *P=*0.056 and EMD: 4563; 95%CI: 1479, 9447 steps count per day; *P=*0.011). Increases in TUG (EMD: -0.50; 95%CI: -0.17, -0.82 sec; *P=*0.005) and TS performance (EMD: 2.75; 95%CI: 0.70, 4.8 reps; *P=*0.012) were also observed in ET.

**Table 2:** Effects of a 12-week, home-based, exercise training program on systemic inflammation, body composition, physical activity levels, functionality, aerobic capacity, cardiometabolic risk factors, disease and health-related quality of life parameters in childhood-onset Takayasu Arteritis patients.

	Control (n=5)		Exercise training (n=9)		Δ (95% CI)ET *vs.* N-ET)	*P*
	PRE	POST	PRE	POST		
CRP (mg/L)	4.72 (7.61)	4.40 (8.77)	3.01 (2.80)	2.17 (2.65)	-0.52 (3.20 to -4.25)	0.764
ESR (mm/h)	4.40 (2.07)	3.40 (1.14)	11.2 (11.1)	7.89 (8.92)	-2.33 (7.24 to -11.9)	0.605
IFN-γ (pg/mL)	1.38 (1.01)	2.20 (2.06)	12.2 (16.8)	5.36 (8.21)	-7.72 (9.45 to -24.9)	0.346
IL-10 (pg/mL)	16.1 (21.9)	8.41 (4.51)	15.8 (15.8)	11.6 (10.2)	3.55 (24.9 to -17.8)	0.723
IL-12p70 (pg/mL)	2.75 (0.48)	3.73 (1.57)	6.24 (3.43)	4.73 (2.89)	-2.49 (0.99 to -5.98)	0.754
IL-1ra (pg/mL)	110.7 (173.4)	169.9 (294.7)	54.6 (35.2)	51.9 (34.6)	-61.8 (28.0 to -151.8)	0.159
IL1-β (pg/mL)	1.78 (0.56)	2.70 (2.43)	3.11 (1.45)	2.26 (1.27)	-1.76 (0.03 to -3.57)	0.053
IL-6 (pg/mL)	12.9 (18.0)	19.7 (34.0)	15.5 (17.6)	30.6 (63.7)	8.31 (68.0 to -51.3)	0.766
TNF-α (pg/mL)	15.7 (8.33)	13.9 (2.93)	14.3 (8.35)	14.2 (9.01)	1.77 (12.3 to -8.79)	0.720
VEGF (pg/mL)	5.50 (9.04)	1.75 (0.94)	1.76 (0.80)	1.39 (0.45)	3.39 (-3.10 to 9.89)	0.277
PDGF (pg/mL)	44.5 (44.8)	46.9 (28.8)	901.8 (1704.2)	381.0 (663.6)	-523.2 (1.256 to -2.303)	0.533
Body mass (kg)	58.7 (13.5)	59.1 (14.5)	55.1 (9.70)	54.6 (9.41)	-0.96 (1.27 to -3.20)	0.367
Fat mass (kg)	13.0 (5.30)	13.1 (5.75)	18.6 (6.10)	18.1 (5.02)	-0.53 (2.05 to -3.11)	0.661
Percentage fat mass (%)	22.2 (6.30)	22.0 (5.80)	33.3 (7.22)	32.4 (6.99)	-0.75 (1.57 to -3.07)	0.494
Visceral fat (g)	242.4 (165.8)	244.4 (173.4)	274.3 (130.1)	253.7 (110.5)	-0.22 (-0.01 to -0.44)	0.040*
Sedentary behavior (%/day)	77.0 (7.08)	82.5 (6.29)	74.4 (9.68)	73.7 (8.91)	-5.24 (0.50 to -11.0)	0.007
Light-intensity PA (%/day)	22.5 (8.15)	17.0 (5.88)	24.1 (9.43)	25.3 (8.89)	6.82 (13.8 to -0.22)	0.056
MVPA (%/day)	0.89 (0.48)	0.99 (0.58)	0.60 (0.30)	0.51 (0.32)	0.07 (0.39 to -0.24)	0.621
Steps (counts/day)	7.299 (4.257)	6.348 (3.097)	8.307 (5.126)	12.890 (6.142)	5.463 (1.479 to 9.447)	0.011*
Timed up-and-go (sec)	5.40 (0.40)	5.52 (0.24)	5.58 (0.40)	5.20 (0.25)	-0.50 (-0.17 to -0.82)	0.005*
Timed-stands (reps)	16.2 (2.00)	16.0 (1.50)	16.8 (1.35)	19.39 (1.36)	2.75 (0.70 to 4.80)	0.012*
Fasting glucose (mg/dL)	79.4 (9.71)	80.0 (8.80)	84.6 (9.14)	82.0 (5.77)	-3.26 (3.77 to -10.31)	0.332
Fasting insulin (mg/dL)	10.3 (5.30)	13.17 (8.1)	11.6 (4.62)	11.8 (4.87)	-2.27 (2.79 to -7.33)	0.347
Total cholesterol (mg/dL)	142.2 (20.6)	149.4 (18.8)	146.3 (37.3)	140.8 (38.3)	-12.7 (8.16 to -33.6)	0.208
HDL cholesterol (mg/dL)	47.4 (10.8)	54.5 (25.04)	61.4 (17.0)	63.8 (18.5)	-4.55 (9.26 to -18.3)	0.486
LDL cholesterol (mg/dL)	79.2 (24.7)	76.8 (30.27)	74.3 (25.4)	66.6 (25.4)	-5.26 (13.4 to -23.9)	0.550
VLDL cholesterol (mg/dL)	15.6 (2.70)	18.2 (4.10)	15.9 (3.52)	19.3 (5.25)	0.84 (7.38 to -5.69)	0.783
Triglycerides (mg/dL)	67.0 (23.4)	93.6 (38.4)	72.1 (27.7)	85.2 (30.2)	13.4 (21.9 to 48.8)	0.422
ITAS score (a.u.)	0.00 (0.00-0.50)	0.00 (0.00-0.50)	0.00 (0.00-0.50)	0.00 (0.00-0.00)	0.00 (0.00-0.00)	0.157
PVAS score (a.u.)	0.00 (0.00-1.00)	0.00 (0.00-1.00)	0.00 (0.00-0.50)	0.00 (0.00-0.00)	0.00 (0.0-0.00)	1.000
VDI score (a.u.)	2.00 (2.00-2.50)	2.00 (1.50-2.00)	1.00 (2.00-2.50)	1.00 (1.00-2.50)	1.00 (1.00-2.50)	0.317
Physical component summary SF-36 (a.u.)	70.0 (60.0-82.3)	86.2 (61.8-94.3)	66.2 (51.2-84.3)	85.0 (69.3-86.8)	–	0.012*
Mental component summary SF-36 (a.u.)	82.7 (58.7-92.7)	84.7 (61.7-91.8)	79.7 (53.2-88.1)	67.0 (55.2-85.8)	–	0.575

Data are expressed as mean (SD), median (IQR 25-75), estimated confidence interval for delta change for the difference between (post-pre) (95% confidence interval [95% CI]) and level of significance (p) between delta change (exercise vs. no exercise training). * means P ≤ 0.05, group difference. CRP, C-reactive protein; ESR, erythrocyte sedimentation rate; IFN-g, interferon gamma; IL-10, interleukin 10; IL-12p70, interleukin 12p70; IL-1ra, interleukin 1 receptor antagonist; IL-1b, interleukin beta; IL-6, interleukin 6; TNF-a, tumor necrosis factor alfa; VEGF, vascular endothelial growth factor; PDGF, platelet-derived growth factor; PA, physical activity; MVPA, moderate-vigorous physical activity; HDL, high-density lipoprotein; LDL, lowdensity lipoprotein; VLDL, very-low-density lipoprotein; ITAS, indian takayasu's arteritis activity score 2010; PVAS, Pediatric Vasculitis Activity Score; VDI, vascular damage index.

Data on disease-related and health-related quality of life parameters were depictured in **Table 2**. Greater increases in physical component summary SF-36 scale were observed in ET *vs.* control (*P=*0.012). Changes in disease-related parameters did not differ between groups (*P*>0.05).

**Figure 2:**
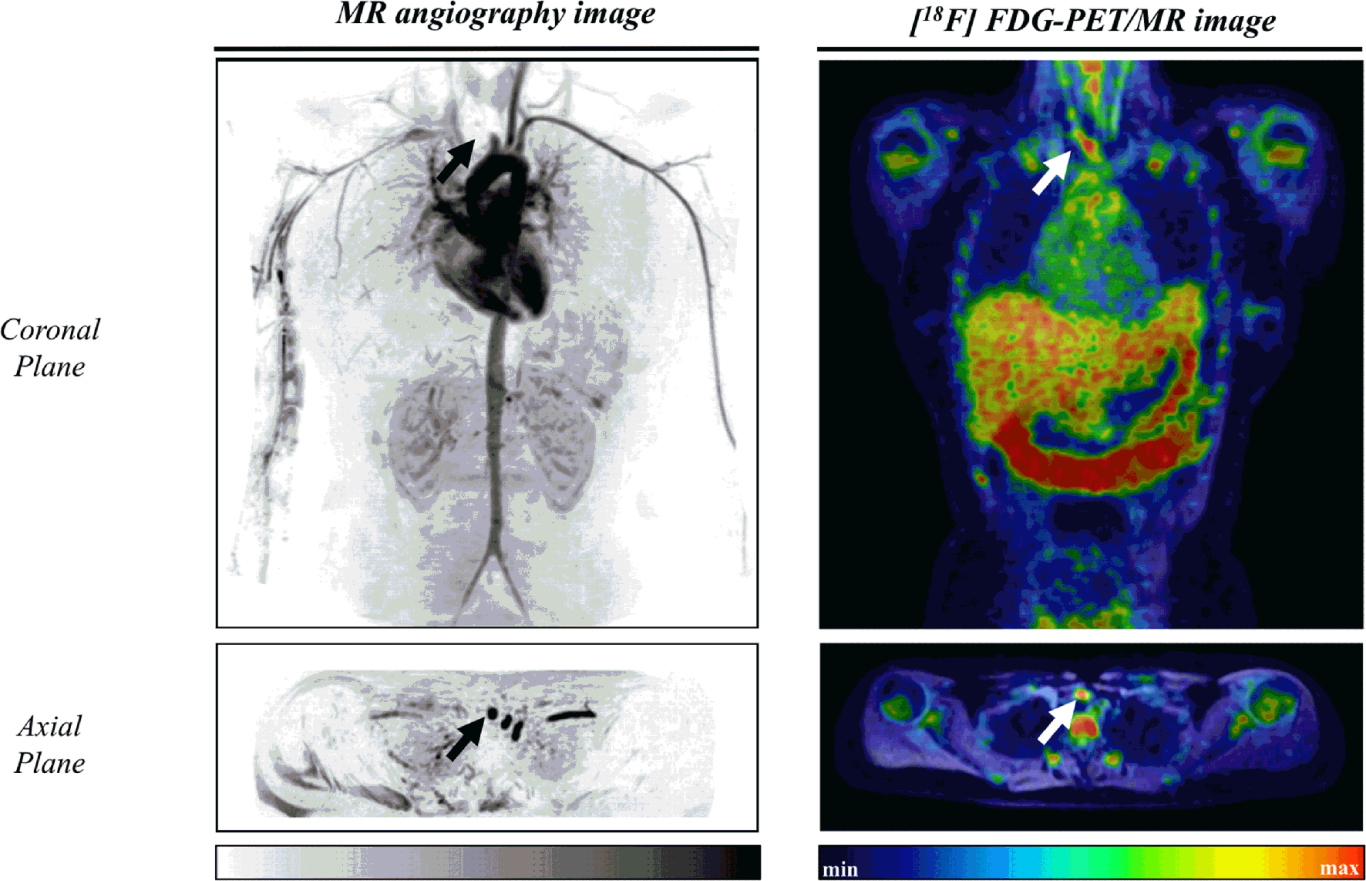
Illustrative case of a 17-year-old female patient with childhood-onset Takayasu Arteritis. Left: Angioresonance of aorta and its branches after intravenous administration of gadolinium. Arrows indicates an occlusion at the third portion of the brachiocephalic trunk. Right: Arterial inflammation grade III as assessed by ^18^F-fluorodeoxyglucose-positron emission tomography/magnetic resonance image at the brachiocephalic trunk (SUV_max_=2.38).

**Figure 3:**
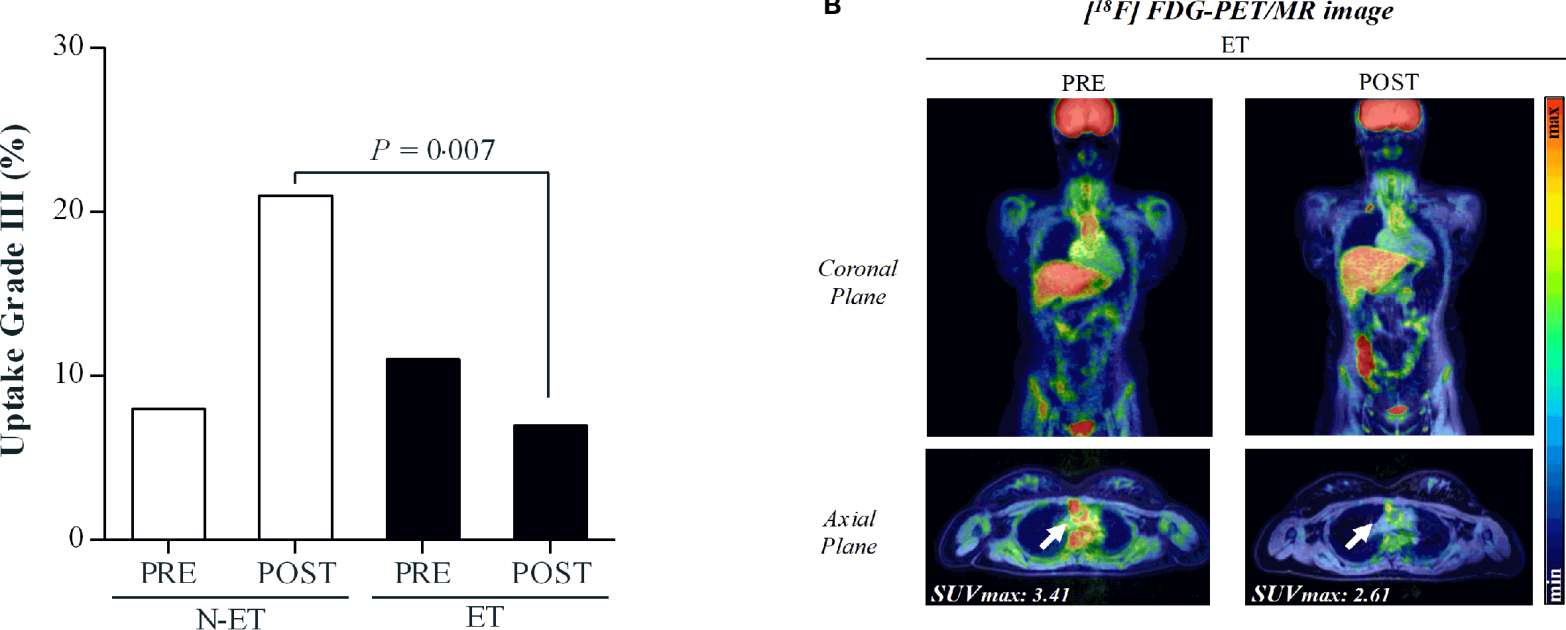
Panel **(A)**: Distribution of vessel segments in grade III, representing severe inflammation. *P*=0.007 for between-group comparison at POST. Panel **(B)**: Illustrative case of a 20-year-old patient with childhood-onset Takayasu Arteritis. The [^18^F] FDG PET/MR imaging revealed grade III inflammation in the ascending aorta artery before the intervention (SUV_max_=3.41). Following the exercise training program, the uptake decreased to grade II (SUV_max_=2.61).

Data regarding arterial inflammation are displayed in **Figures 2**, **3A, B** and **Table 3**. After the intervention, ET showed a lower frequency of vessel segments with severe inflammation (grade III) as compared with control group (*P*=0.007; **Figure 3**, panel A). ET and control showed comparable FDG uptake assessed by SUV_max_ in the 19 vessels assessed before and after the intervention (**Table 3**).

**Table 3:** Effects of a 12-week, home-based, exercise training program on SUV_max_, assessed by [^18^F] FDG-PET/MRI, in childhood-onset Takayasu arteritis patients.

	Control (n)		Exercise training		Δ (95% CI) ET *vs.* N-ET)	*P*
	PRE	POST	PRE	POST		
SUV_max_ liver	2.46 (0.47)	2.22 (0.21)	2.82 (0.44)	2.75 (0.35)	0.16 (-0.25 to 0.58)	0.411
Right carotid artery	1.82 (0.28)	2.20 (0.70)	1.91 (0.18)	1.73 (0.28)	-0.55 (0.08 to -1.18)	0.080
Left carotid artery	1.99 (0.12)	2.45 (1.22)	1.76 (0.16)	1.72 (0.28)	-0.50 (0.45 to -1.46)	0.270
Right vertebral artery	1.54 (0.41)	1.63 (0.17)	1.82 (0.18)	1.53 (0.40)	-0.37 (0.04 to -0.79)	0.070
Left vertebral artery	1.68 (0.53)	1.64 (0.47)	1.68 (0.15)	1.67 (0.42)	0.01 (-0.65 to 0.69)	0.955
Ascending aorta	2.39 (0.37)	2.47 (0.25)	2.73 (0.44)	2.64 (0.34)	-0.16 (0.43 to -0.75)	0.549
Aortic arch	1.80 (0.18)	1.87 (0.18)	2.48 (0.28)	2.63 (0.41)	0.08 (-0.53 to 0.69)	0.777
Descending aorta	1.95 (0.33)	1.91 (0.16)	2.21 (0.29)	2.22 (0.27)	0.04 (-0.30 to 0.39)	0.798
Brachiocephalic artery	1.72 (0.27)	1.95 (0.35)	2.09 (0.40)	2.19 (0.35)	-0.12 (0.31 to -0.56)	0.538
Right subclavian artery	1.33 (0.44)	1.48 (0.29)	1.63 (0.40)	1.74 (0.51)	-0.03 (0.68 to -0.76)	0.907
Left subclavian artery	1.37 (0.23)	1.74 (0.57)	1.67 (0.40)	1.74 (0.40)	-0.31 (0.35 to -0.97)	0.321
Pulmonary trunk	2.47 (0.98)	1.88 (0.23)	2.32 (0.67)	2.05 (0.39)	0.31 (-0.73 to 1.36)	0.521
Celiac trunk	1.38 (0.21)	1.48 (0.14)	2.21 (0.54)	1.99 (0.63)	-0.31 (0.47 to -1.10)	0.395
Superior mesenteric artery	1.48 (0.18)	1.47 (0.19)	1.69 (0.37)	1.74 (0.36)	0.06 (-0.42 to 0.55)	0.769
Abdominal aorta	1.75 (0.18)	1.67 (0.18)	2.35 (0.45)	2.27 (0.28)	0.01 (-0.33 to 0.34)	0.962
Right renal artery	3.58 (2.35)	3.61 (2.19)	2.29 (1.35)	2.36 (1.27)	0.05 (-2.32 to 2.42)	0.963
Left renal artery	2.01 (0.81)	1.99 (0.36)	2.96 (3.25)	4.79 (5.29)	1.85 (1.74 to 5.45)	0.278
Inferior mesenteric artery	1.30 (0.26)	1.42 (0.26)	1.56 (0.59)	1.50 (0.23)	-0.17 (0.31 to -0.67)	0.441
Right iliac artery	1.85 (0.62)	1.82 (0.26)	2.44 (0.64)	1.87 (0.45)	-0.41 (0.70 to -1.54)	0.419
Left iliac artery	1.55 (0.34)	1.59 (0.34)	1.78 (0.35)	1.72 (0.38)	-0.03 (0.39 to -0.46)	0.856

Data are expressed as mean (SD). Estimated a confidence interval for delta change for the difference between (post-pre) (95% confidence interval [95% CI]) and level of significance (P) between delta change (exercise vs. no exercise training).

## Discussion

To our knowledge, this is the first RCT involving c-TA patients, and the only one that investigated the effects of exercise in pediatric with this disease. The main finding of this study was that exercise was safe and did not exacerbate arterial inflammation. Exercise also appeared to promote clinical benefits, including reduction in visceral fat, improvement in muscle function, and increases in spontaneous physical activity and physical component summary SF-36 scale. Collectively, these results demonstrate that exercise may play a therapeutic role in the treatment of patients with c-TA.

TA is a rare, primary vasculitis that predisposes patients to a high risk of mortality and morbidities, including vascular stenosis, occlusion, aneurysm formation, ischemia, stroke, arterial hypertension, and heart failure (2). Patients with rheumatologic pediatric diseases are often hypoactive, which may worsen their clinical symptoms, fitness and physical function, which, in turn, predispose them to a more inactive lifestyle (26, 27). We have argued that this vicious circle should be “disrupted” by incorporating physical activity into the patients’ routines (28), thereby alleviating the main clinical manifestation of the disease and improving cardiovascular health.

This hypothesis was put forward in this RCT, whereby an exercise program was delivered to c-TA patients, with the main hypothesis that exercise would be clinically beneficial, without increasing arterial inflammation, considered the main pathophysiologic feature of this disease (5). [^18^F] FDG PET/MRI is highly effective in assessing the activity and the extent of large-vessel vasculitis, and visual grading is validated as representing the severity of inflammation (29). This method was shown to be highly sensitive to detect subtle change in disease activity in TA, with the intensity of FDG accumulation decreasing in response to therapy (17, 30). This makes [^18^F] FDG particularly useful for monitoring sub-clinical inflammatory responses in TA since, in a substantial number of patients with signs and symptoms of inflammation in the vessels, no changes were seen in serum levels of acute phase reactants (e.g., erythrocyte sedimentation rate and C-reactive protein), or disease activity assessed by disease-specific tools (e.g., ITAS) (31). Therefore, using this robust imaging technique, we provided compelling evidence that exercise did not exacerbate arterial inflammation, as expected (see **Figure 3** and **Table 3**). In fact, exercised patients had a lower frequency of vessels with severe inflammation (grade III) as compared with controls. Interestingly, some exercised patients did show reduced inflammation in several vessels segments (see **Table 3**). Similar reports of reduced [^18^F] FDG accumulation in TA patients receiving immunosuppressant therapy have been published, but these are fundamentally diagnosis studies, enrolling low number of patients (n = 1 to 4) on open-label, heterogeneous therapies (30, 31). This hampers any direct comparison between our data and others. Although it was clear that exercise did not increase arterial inflammation in this study, caution should be taken in concluding that physical exercise led to an anti-inflammatory effect, since the sample size was limited, particularly in the control group, and the proportion of vessels with severe inflammation were apparently different between groups at baseline, despite the lack of a statistical significance.

**Figure 4:**
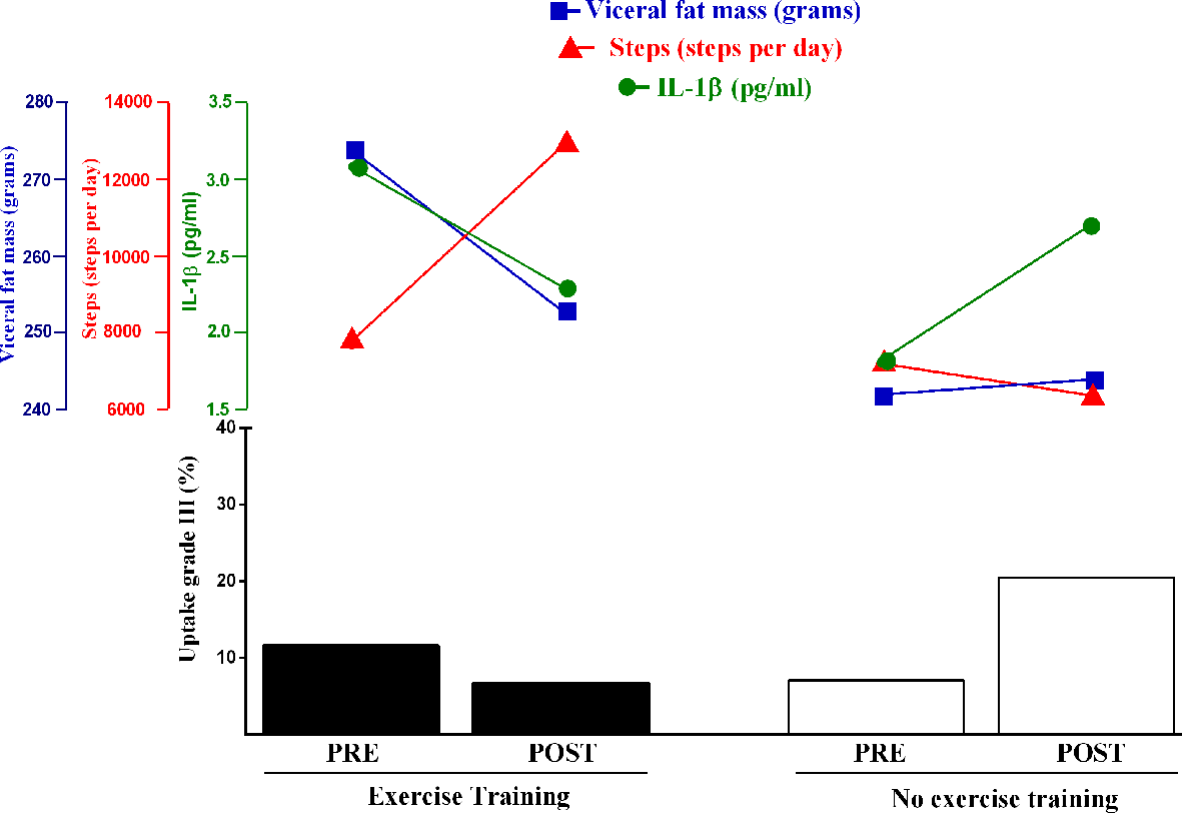
Overview of the changes in severe arterial inflammation, visceral fat mass, IL1-β, and number of steps following a 12-week, home-based, exercise training program or control (no exercise) in c-TA patients. Noticeably, the exercise-induced amelioration in severe arterial inflammation was mirrored by decreases in visceral fat and IL-1β levels (tendency towards significance) and increases in physical activity. The hypothesis raised by this study that these responses might be interrelated should be tested by larger trials.

Of relevance, exercise improved muscle strength and greatly increased the number of steps per day. One may hypothesize that improvements in functionality could have predisposed the patients to a more active lifestyle, possibly leading to improvements in visceral fat, with possible influence on some inflammatory markers, such as IL1-β (tendency towards significance), an important mediator of the inflammatory response (12, 32, 33). Noticeably, physical component summary SF-36 was also improved following exercise, indicating that exercise can also impact some aspects of quality of life. Overall, these outcomes suggest that physical exercise can be therapeutically beneficial in c-TA (see **Figure 4** for an overview of the main findings). While further studies are necessary to confirm the efficacy of exercise in c-TA as previously discussed (also see the limitations below), numerous clinical, imaging, and laboratorial markers did not reveal any evidence of disease flaring or any adverse events, meaning that exercise can be a safe intervention in this disease.

The main strengths of this study are the use of a randomized controlled design and the application of a comprehensive battery of tests, including some ‘gold-standard’ techniques (notably [^18^F] FDG PET/MRI), to explore the effects of exercise for the first time in this disease. However, this study is not without limitations. First, in spite of enrolling patients from three different centers, sample size was still limited. As previously stated, nonetheless, we recruited all the available patients in the three specialized ambulatory care services managing patients with c-TA in Sao Paulo, the most populous city in the Southern Hemisphere, which might lessen criticisms regarding the representativeness of our sample. In addition, to mitigate the lack of power typically inherent to small-scale trials, we opted for a 2:1 randomization and a case-complete analysis; however, we recognized that these measures have disadvantages, particularly when missing-data mechanism is not completely at random. In addition, patients’ characteristics (e.g., age, sex, treatment) were relatively heterogeneous, and sensitivity analyses including specific sub-groups were not possible because of the limited sample size. In this regard, one may argue that groups were unbalanced for sex. However, controlling for sex in a *post-hoc* analysis, we observed similar results overall, with some tendencies actually reaching statistical significance (i.e., IL1-β and light-intensity physical activity; *P*=0.05 and *P*=0.01, respectively), despite the increased uncertainty for visceral fat (*P*=0.12). As the sample size was low and unbalanced owing to the adopted 2:1 randomization approach, we cannot rule out the possibility that other variables, such as the medication regimen, may have influenced the results. Moreover, our patients were in remission according to the clinical assessments; therefore, these findings cannot be extrapolated to patients with a more active disease. Multinational studies involving larger samples and intention-to-treat analysis should be conducted to overcome these limitations and confirm the current findings. Finally, the home-based, exercise intervention was especially tailored for our population; therefore, its cross-cultural replicability needs confirmation and potential adjustments.

In conclusion, a home-based, exercise training program was shown to be safe as it did not exacerbate arterial inflammation or cause any adverse event in c-TA patients. Of relevance, physical exercise appeared to improve visceral fat, muscle function, and physical activity levels, providing novel evidence that exercise could be considered as a therapeutic tool to improve general health in patients with c-TA. Larger trials should confirm the value of exercise for c-TA.

## Data Availability Statement

The original contributions presented in the study are included in the article/supplementary material. Further inquiries can be directed to the corresponding author.

## Ethics Statement

The studies involving human participants were reviewed and approved by Comissão de Ética para Análise de Projetos de Pesquisa (CAPPesq) and by the Plataforma Brasil registered in May/2017 under the number 2.070.921. Written informed consent to participate in this study was provided by the participants’ legal guardian/next of kin.

## Author Contributions

CA: Conceptualization, methodology, formal analysis, investigation, writing – original draft, visualization, and funding acquisition. GC: Conceptualization, methodology, writing – original draft, visualization, and funding acquisition. MT: Conceptualization, investigation, and funding acquisition. CC: Methodology, formal analysis, and investigation. ML: Conceptualization, methodology, formal analysis, investigation, and writing – original draft. CB: Conceptualization, methodology, and resources. HF: Methodology and resources. AP: Methodology, writing – review, and editing. CS: Conceptualization, writing – review, and editing. LC: Methodology, writing – review, and editing. NA: Methodology and investigation. SG: Formal analysis, investigation, writing - original draft, and visualization. RP: Conceptualization, investigation, funding acquisition, resources, writing – review, and editing. HR: Writing - review and editing. BG: Conceptualization, methodology, writing – original draft, supervision, project administration, and funding acquisition. All authors contributed to the article and approved the submitted version.

## Funding

This study was supported by grants from CA and BG were supported by São Paulo Research Foundation – FAPESP (grants 2017/07358-9 and 2017/13552-2) and was also financed in part by the Sociedade Brasileira de Reumatologia (SBR). Conselho Nacional de Desenvolvimento Científico e Tecnológico (CNPq 303422/2015-7 to CS), Fundação de Amparo à Pesquisa do Estado de São Paulo – FAPESP (grants # 2015/03756-4 to CS) and by Núcleo de Apoio à Pesquisa “Saúde da Criança e do Adolescente” da USP (NAP-CriAd) to CS.

## Conflict of Interest

The authors declare that the research was conducted in the absence of any commercial or financial relationships that could be construed as a potential conflict of interest.

## Publisher’s Note

All claims expressed in this article are solely those of the authors and do not necessarily represent those of their affiliated organizations, or those of the publisher, the editors and the reviewers. Any product that may be evaluated in this article, or claim that may be made by its manufacturer, is not guaranteed or endorsed by the publisher.

## Abbreviations

TA, Takayasu arteritis; c-TA, childhood-onset Takayasu arteritis; ITAS2010, the Indian Takayasu Arteritis Score 2010; PVAS, the Paediatric Vasculitis Activity Score; VDI, vasculitis Damage Index; VEGF, vascular endothelial growth factor; TUG, timed up-and-go; TS, timed stands; [^18^F] FDG, 18 fluoro-deoxy-D-glucose; PET/MRI, positron emission tomography with magnetic resonance imaging; SUV, standard uptake value; MVPA, moderate-vigorous physical activity.
